# CONFLICTING IMAGES OF AGING IN JAPAN: WHY DON’T PEOPLE WISH TO CELEBRATE THEIR 100TH BIRTHDAY?

**DOI:** 10.1093/geroni/igae098.1019

**Published:** 2024-12-31

**Authors:** Saori Yasumoto, Takeshi Nakagawa, Yukie Masui, Yasuyuki Gondo

**Affiliations:** Osaka University, Suita, Osaka, Japan; National Center for Geriatrics and Gerontology, Obu, Aichi, Japan; Tokyo Metropolitan Institute of Gerontology, Tokyo, Japan; Osaka University, Suita, Osaka, Japan

## Abstract

Japan is a country known for its longevity. While people practice customs to celebrate old age, negative stereotypes and discrimination against older people exist as well. This presentation discusses how people perceive aging and longevity in a society where such conflictive images exist. The research on the perception of aging is essential because it impacts older people’s physical and psychological well-being; however, culturally sensitive research to understand why people perceive aging as such is limited. In this presentation, we introduce the research results from surveys: 1) on age stereotypes conducted by targeting 1,000 people aged between 30 and 75, 2) on wish to live 100 years old by targeting 550 people aged between 77 and 81, and 3) on the longevity aspiration by targeting 33,500 people aged between 20 and 69. We found that people in Japan tend to be pessimistic about becoming old and living a long life. The most common reason was, “I do not want to cause trouble to other people.” Studies conducted outside Japan often indicate that sickness and functional decline make people see aging negatively. In Japan, the socially constructed value of “people become a burden on others due to nursing care” creates a negative image of old age. The number of centenarians is expanding in Japan, whereas people have a negative image of living longer. For us to age happily, there is an urgent need to create a culture where receiving care is not a burden to others.

